# The lateralization accuracy of inferior petrosal sinus sampling in Cushing’s disease: experiences of a tertiary center

**DOI:** 10.55730/1300-0144.5500

**Published:** 2022-08-25

**Authors:** Elif Tutku DURMUŞ, Ayşegül ATMACA, Fatih UZUNKAYA, Ramis ÇOLAK, Buğra DURMUŞ

**Affiliations:** 1 Department of Endocrinology and Metabolism, Faculty of Medicine, Ondokuz Mayıs University, Samsun, Turkey; 2 Department of Radiology, Faculty of Medicine, Ondokuz Mayıs University, Samsun, Turkey

**Keywords:** Cushing’s disease, bilateral inferior petrosal sinus sampling, lateralization, magnetic resonance imaging

## Abstract

**Background/aim:**

The purpose of this study is to determine the accuracy of bilateral inferior petrosal sinus sampling (IPSS) in lateralization and to investigate variables associated with accurate IPSS lateralization prediction.

**Materials and methods:**

Initially, data from 55 patients who underwent IPSS in our institution were reviewed retrospectively. IPSS lateralization and pituitary magnetic resonance imaging (MRI) results of these patients were compared with postoperative follow-up and immunohistochemical data to calculate the positive predictive values (PPVs) for IPSS and MRI. Variables likely to be associated with the accurate prediction of IPSS lateralization were analyzed.

**Results:**

Twenty-seven patients (85.2% female, mean age of 38.5 ± 13.1 years) were enrolled in the study. With IPSS, interpetrosal ratios were found to be ≥ 1.4 in 26 (96.2%) cases, and this ratio correctly predicted adenoma localization for 18 patients (PPV: 69.2%). For 16 (59.2%) patients, right lateralization was detected, while left lateralization was detected for 10 (37%) patients. Right-sided IPSS lateralization was associated with enhanced accuracy (p = 0.026). No masses were detected in the MRI images of 10 (37%) patients, while microadenoma of ≤ 6 mm was detected for 17 (63%) patients. MRI results (when positive) correctly identified adenoma localization for 14 of the patients with lateralization accuracy higher than that of IPSS (PPV: 82.3% vs. 69.2%).

**Conclusion:**

IPSS is a valuable procedure in detecting tumor lateralization, especially in patients with Cushing’s disease who have negative pituitary MRI results. However, since lateralization has a limited reliability, the pituitary gland should be comprehensively evaluated by taking into account the MRI findings (if positive) as well as data on the side of IPSS lateralization.

## 1. Introduction

Cushing’s disease (CD), the most common cause of adrenocorticotropic hormone (ACTH)-dependent Cushing’s syndrome (CS), generally results from ACTH-secreting pituitary tumors, but in rare cases, it may also be caused by ectopic (nonpituitary) ACTH-secreting tumors [1]. The presence of similar clinical and laboratory findings for these two conditions may complicate the differential diagnosis. Further factors complicating the diagnostic process are as follows: roughly 40% of all ACTH-secreting pituitary tumors are not revealed by imaging methods and it is estimated that ≥10% of all individuals in the general population have incidental microadenomas with small size and lack of clinical symptoms and signs [2]. This complex situation has made bilateral inferior petrosal sinus sampling (IPSS) the gold standard to distinguish between pituitary and ectopic causes [1]. ACTH levels measured by IPSS simultaneously from the inferior left and right petrosal sinuses and peripheral venous drainage can be directly compared with each other [1, 3]. Based on previous studies, CD may be diagnosed in the presence of baseline central-to-peripheral (C/P) gradient ratios above 2 or 3 following the stimulation of corticotropin-releasing hormone (CRH) and ectopic tumors secreting ACTH can then be confidently ruled out [1, 4].

IPSS is also used to ascertain on which side the adenoma is localized in cases of CD, and it can guide the surgeon during the procedure to be performed [1, 4]. Previous studies reported that the presence of an ACTH gradient difference of at least 1.4 between the inferior petrosal sinuses (i.e. comparing the two sides) can be used to predict the lateralization of adenoma in CD [3–5]. However, although IPSS has a high diagnostic accuracy for the localization of the pituitary gland, its reliability for tumor lateralization is still controversial. Sometimes, IPSS lateralization may be incompatible with true tumor localization due to the effects of venous plexus drainages or asymmetrical anatomy. The probability that lesions in CD may be very small and the limited number of tests that we can perform preoperatively also reveal the need for other predictors of lateralization accuracy in IPSS. However, the number of studies conducted on such predictive factors that may contribute to surgical remission remains very limited in the literature.

This study was planned to determine the accuracy of IPSS in the lateralization of pituitary adenoma, compare that accuracy with the accuracy of positive pituitary magnetic resonance imaging (MRI) findings, and evaluate whether there is a need for other factors predicting the lateralization accuracy of IPSS by taking advantage of our experiences with IPSS in our institution.

## 2. Materials and methods

### 2.1. Study design and participants

In this study, we retrospectively evaluated the data of 55 patients diagnosed with ACTH-dependent CS between 2010 and 2019 who underwent IPSS for differential diagnosis in the interventional radiology department of our institution. All patients met the diagnostic criteria for CS as given in the clinical practice guidelines of the Endocrine Society [6]. Twenty-eight patients were excluded from the study, 3 of whom were diagnosed with ectopic CS according to IPSS results, 12 of whom were not followed after the decision for an operation due to the diagnosis of CD, 8 of whom were not operated on due to follow-up decisions regarding subclinical CS, 4 of whom preferred an external medical center for their operations, and 1 of whom was not followed after the operation. Thus, 27 patients were enrolled in the study, all of whom had a diagnosis of CD as confirmed by IPSS, underwent transsphenoidal hypophysectomy in our hospital, and admitted regularly for their follow-up examinations. Standard diagnostic test results for patients with CS [serum cortisol (μg/dL), serum ACTH (pg/mL), midnight serum cortisol (μg/dL), 24-h urinary free cortisol (UFC), dehydroepiandrosterone sulfate (DHEAS), 1 mg dexamethasone suppression test (DST), low-dose 2-day dexamethasone test (LDDT), and high-dose (8 mg) DST)] were recorded. All patients were assessed with pituitary MRI before IPSS. IPSS indications were either a normal pituitary MRI result or adenoma of ≤6 mm in patients biochemically diagnosed with ACTH-dependent CS [1]. The IPSS procedures were performed in the Interventional Radiology Department of our institution. All patients were comprehensively informed about the risks, benefits, and potential complications of the IPSS procedure before it was performed and informed consent was obtained from the patients before the procedure. CRH was administered to all patients during IPSS. Central/peripheral (C/P) ratios for ACTH levels were checked at the beginning, during IPSS, and after CRH injection, and right/left (R/L) ratios for ACTH levels before and after CRH administration were recorded. The ACTH ratios were calculated for peripheral venous blood samples from the left and right inferior petrosal sinuses at all time points (C/P values). IPSS results were accepted as providing a positive diagnosis for pituitary production of ACTH if the peak ratio measured after administering CRH was >3 [4]. Interpetrosal gradient ratios were additionally determined between the two petrosal sinus samples obtained at each considered time point and these ratios were taken to be predictors of lateralization at values of >1.4 [4–6]. Before undergoing operation, all patients were evaluated with clinical and preoperative imaging findings and IPSS results during meetings of the pituitary council, which were also attended by endocrinology, neurosurgery, endocrine pathology, and neuroradiology specialists, as well. According to the evaluation performed during the meeting of this council, patients were operated on by a neurosurgeon experienced in pituitary surgery, who performed adenomectomy and/or hemihypophysectomy using the transsphenoidal microsurgery technique. Those with postoperative serum cortisol concentrations of <2 μg/dL were considered to be in remission and glucocorticoid replacement was performed during their follow-up for treatment of the hypothalamic-pituitary-adrenal (HPA) axis [1, 7]. The exact location of the mass was determined by evaluating the immunohistochemical and postoperative follow-up results (remission/persistent disease) of the patients, and those results were then compared with the preoperative IPSS lateralization and pituitary MRI results. The study was granted approval by the relevant institutional ethics committee (OMU KAEK 2019/480).

### 2.2. Assay

Cortisol levels were measured by competitive electrochemiluminescent immunometric assay using the Cobas 8000 Modular Analyzer Series (Roche Diagnostics). For the cortisol assay, analytical sensitivity was 0.018 μg/dL and the intraassay coefficient of variation (CV) was 1.5%–1.7%. ACTH and DHEAS levels were measured by solid-phase two-site sequential and competitive chemiluminescent immunometric assays, respectively, using the IMMULITE 2000 Immunoassay System (Siemens Healthcare Diagnostics). For the ACTH assay, analytical sensitivity was 5 pg/mL, intraassay CV was 8.7%–9.5%, and interassay CV was 6.1%–10%. For the DHEAS assay, analytical sensitivity was 3 μg/dL, intraassay CV was 4.9%–9.8%, and interassay CV was 7.9%–13%. After urine extraction with dichloromethane, 24-h UFC was measured by the electrochemiluminescent technique with intra- and interassay CVs of 1.5%–1.7% and 1.8%–2.2%, respectively.

### 2.3. Imaging protocol

MRI examinations were performed using the same pituitary MRI protocol for all patients with a 1.5-T Siemens MR unit (Magnetom Symphony Quantum, Siemens Medical Systems) or 1.5-T Philips MR unit (Achieva; Philips Healthcare). T1-weighted and T2-weighted spin echo sequences were obtained with a slice thickness of 3 mm for the coronal and sagittal planes. Later, T1-weighted turbo spin echo dynamic sequences were obtained with 3-mm slice thickness for the coronal and sagittal planes after gadolinium injection (0.1 mmol/kg).

### 2.4. Catheterization protocol

Under ultrasonographic guidance, 5-Fr introducer sheaths were placed in both common femoral veins. Subsequently, 2500 U of heparin was administered intravenously to avoid thrombotic complications. Habitually starting from the right side, a vertebral shaped 4-Fr diagnostic catheter was advanced over a hydrophilic guidewire to cannulate the orifice of the ipsilateral inferior petrosal sinus using the internal jugular vein route. By hand injection of iodinated contrast medium, the anatomy of the right inferior petrosal and cavernous sinuses as well as that of the intercavernous sinus were revealed with the patient in supine position, and fluoroscopic images were recorded. The same procedure was thereafter conducted for the left side (Figure). In the event of failure to cannulate any orifice, contralateral road-mapping was performed to discover the entry point. Right or left oblique projections were used if this action did not make cannulation possible. Upon catheterization of the bilateral inferior petrosal sinuses, a blood sample of 2 mL was simultaneously taken at 0 min from both catheters as well as from the introducer sheath on the right or left side to measure basal ACTH levels. Simultaneous samplings were repeated at 3, 5, 8, 10, and 15 min following intravenous CRH injection (1 μg/kg up to 100 μg maximal dose). Blood samples were stored in pre-labeled EDTA tubes and sent to the laboratory on ice. These procedures were ended with bilateral groin hemostasis following the withdrawal of the catheters.

### 2.5. Statistical analysis

While analyzing descriptive statistics, continuous variables were given as median [with range or interquartile range (IQR)], while categorical variables were given as frequency (with percentage). The normality of the data was evaluated with the Kolmogorov-Smirnov test. Independent categorical variables were compared using chi-square or Fisher’s exact tests, while dependent categorical variables were compared using the McNemar test. Nonparametric data were compared using the Mann-Whitney U test. In the process of determining the rates of lateralization accuracy, positive predictive values (PPVs) were obtained with the following calculation for the interpetrosal gradient as measured by IPSS and pituitary MRI results: count of true positive results obtained for the test /(count of true positive results + count of false positive results). All statistical comparisons were two-tailed and values of p < 0.05 were considered to be statistically significant.

## 3. Results

Of the 27 patients enrolled in this study, 23 (85.2%) were female and 4 (14.8%) were male, with a mean age of 38.5 ± 13.1 (18–65) years and a mean follow-up period of 42.5 ± 30.4 months. At the time of diagnosis, the median serum cortisol level of the patients was found to be 21.9 (13.0–25.8) μg/dL (normal range: 6.2–19.4 μg/dL), while median plasma ACTH was 65.8 (45.4–96.0) pg/mL (normal range: < 46 pg/mL), and low-dose 2-day DST results could not be suppressed for any patients. All preoperative endocrinological test results of the patients, obtained prior to the bilateral IPSS results, are shown in Table 1.

For all patients, C/P ratios obtained by IPSS were >2 at baseline and >3 after CRH injection. Interpetrosal ratios were ≥1.4 for 26 patients (96.2%), while it was <1.4 for 1 patient and lateralization could not be detected (Table 2). IPSS correctly predicted the side of the pituitary gland that contained the adenoma in 18 (69.2%) of these 26 patients with lateralization, while the adenoma was located contralaterally in 8 cases (30.8%) (Table 3). According to these results, PPV was considered to be 69.2% for lateralization in IPSS. IPSS lateralized the right side in 16 (59.2%) cases and the left side in 10 (37%) cases. The accuracy rate for right lateralization was found to be significantly higher than that for the left side (p = 0.026) (Table 4). When evaluated based on the CRH stimulation response pattern, 22 (84.6%) patients with lateralization were found to have consistent lateralization (interpetrosal ratio of ACTH levels prior to and after CRH administration of ≥1.4), while 3 patients (11.5%) had lateralization only before CRH administration and 1 (3.8%) patient had lateralization only after CRH administration. The PPV was found to be 68.1% for patients with consistent lateralization, while it was 75% for other CRH stimulation response patterns. The IPSS lateralization accuracy rates for variables other than side of lateralization [pattern of response to CRH, peak interpetrosal gradient ratio, peak interpetrosal gradient ratio time (before or after CRH administration), age, sex, MRI positivity, and ratio 0’ (right/left ratios for ACTH levels prior to CRH administration) and ratio max (right/left ratios of ACTH levels following CRH administration)] were similar to each other (Table 4). No thromboembolic or any other complications associated with the IPSS procedure were observed among our patients.

No mass was detected by pituitary MRI for 10 (37%) patients, while MRI results were positive for adenoma in 17 (63%) cases. For 9 of 10 patients with negative pituitary MRI results, lateralization was detected by IPSS, with IPSS showing the lateralization correctly in 6 (66.7%) of the cases. For the remaining MRI-negative patient, IPSS showed no lateralization due to bilateral adenoma and a total hypophysectomy was performed. For cases in which the results of pituitary MRI suggested the presence of adenoma, the location of the adenoma was accurately revealed by the imaging results of 14 of 17 patients (PPV: 82.3%). Compared to the accuracy of IPSS for lateralization of adenoma, MRI (when positive) had proportionally higher accuracy (PPV: 69.2% vs. 82.3%). By the results of immunohistochemical examinations, 20 (74.1%) patients had corticotropic adenoma and 1 (3.7%) patient had gonadotropic adenoma, while no evidence of pituitary adenoma was obtained for 6 (22.2%) patients. Remission was achieved after surgery for 19 (70.4%) of the patients, while remission could not be achieved in 8 (29.6%) cases. In 3 patients (11.1%), who initially showed remission, recurrence developed during follow-up. Table 3 summarizes the demographic characteristics of these patients, preoperative MRI findings, IPSS lateralization results, surgical and histopathological data, and postoperative follow-up results.

## 4. Discussion

This study has revealed that IPSS had a PPV of 69.2% for lateralization (i.e. right or left side) of tumors in a sample of patients with CD, with a higher rate of accuracy obtained for tumors located on the right side. Additionally, the accuracy rate of positive pituitary MRI results for the localization of these adenomas was found to be 82.3%. Thus, compared to the PPV of IPSS, MRI (when positive) was more accurate in its ability to predict the locations of tumors of the pituitary gland.

Previous studies assumed that asymmetric, hypoplastic, or plexiform petrosal sinus anatomy could cause false lateralization results by IPSS together with catheter positioning and the petrosal collateral venous drainage of the sinuses [3, 5, 8–10]. It is a common belief that, due to the potential impact of all these variables, there may not be a conclusive evidence that an appropriate pituitary blood sample can be drawn from both inferior petrosal sinuses even if the catheters are positioned correctly. In support of this opinion, the accuracy range of lateralization of adenomas in cases of CD by IPSS was reported in a wide range of 48%–84% in the literature [3–5, 11]. Another issue that may contribute to differences in results is the fact that in some studies CD remission alone and in others positive immunohistochemical analysis alone is taken as a basis in determining the accuracy of lateralization. The inclusion of only histopathologically verifiable cases in the study and the exclusion of cases of remission after treatment from evaluation may cause IPSS lateralization accuracy results to seem lower. This is because the lesions in CD can be very small and losses may therefore develop due to surgical intervention. In our study, we determined the lateralization accuracy to be 69.2% with the aim of minimizing selection bias and being able to evaluate lateralization reliability more accurately, while also taking into account the consequences of postoperative remission/persistent disease in immunohistochemical evaluation and follow-up. In a study that enrolled 501 patients with CD with the aim of determining the reliability of IPSS in lateralization by calculating PPV values, similarly to our study, IPSS results accurately revealed which side of the pituitary gland harbored the tumor in 273 cases (69%), while contralateral tumor location was subsequently identified in 123 cases (31%) [3]. Other researchers concluded that the results of bilateral IPSS for pathology-proven cases were consistent with those of pathologic lateralization for approximately 50% of the patients enrolled in the study [11]. In another study, accuracy rates were evaluated among patients for whom surgical lateralization of the tumor was confirmed to be either right or left, disregarding the results of lateralization by IPSS, and a fairly low accuracy rate of 54% was obtained [12].

Due to the limited examination possibilities for accurately determining the localization of adenoma in cases of CD, factors that may predict the accuracy of IPSS lateralization, the reliability of which is still a matter of debate, are important. However, studies in the literature on factors associated with lateralization accuracy are quite limited and give conflicting results. Wind et al. concluded that findings of left-sided IPSS lateralization and concordant lateralization results obtained preceding and following the administration of CRH both correlated with higher levels of accuracy [3]. However, Feng et al. reviewed 51 cases to ascertain whether or not lateralization results obtained by IPSS were similar to those obtained by surgery, and the cases in the group with concordant results were found to have a higher rate of right lateralization in the process of surgical exploration (p = 0.020) [13]. There was a higher frequency of right-sided lateralization in our study, as well, and right-sided lateralization results obtained by IPSS were found to be related to higher rates of accuracy. This could be understood in the context of asymmetrical venous drainage resulting from assorted possible anatomical variations, as noted in previous research [5, 9]. Some studies have revealed inconsistent ACTH concentration values between IPSS results, even among healthy individuals [14]. Accordingly, it is clear that asymmetric venous drainage is a relatively common finding for the bilateral inferior petrosal sinuses and this fact must be considered whenever IPSS is being performed [13]. Studies showing that choosing patients with symmetrical venous drainage is likely to increase the accuracy of lateralization support this claim, as well [5, 9]. It is clear that future studies conducted on this issue while considering venous drainage patterns will give more reliable results.

With pituitary MRI, only about 50% of microadenomas can be clearly shown because most ACTH-secreting pituitary adenomas are very small in size [15]. In addition, the current guidelines are in consensus that IPSS is necessary for all patients with lesions of ≤6 mm or negative MRI results while patients with lesions of ≥10 mm do not need IPSS because a pituitary lesion detected by MRI may be an accidental sellar mass and the source of ACTH may be ectopic [1]. In the literature, however, data on the reliability of MRI positivity in adenoma localization may be affected by the presence of microadenomas being ≤6 mm among the selection criteria for IPSS, with rates consequently detected to be lower than they really are. In the present study, IPSS was less reliable in lateralization than MRI (when positive), with PPVs of 69.2% and 82.3%, respectively. Previous studies have demonstrated findings similar to these regarding the advantages of pituitary MRI over IPSS considering the results for lateralization, with PPVs reported as 86% vs. 69%, 75% vs. 48%, and 70% vs. 63%, respectively [3, 11, 16]. These results support the idea that MRI is likely to have a higher reliability compared to IPSS in cases of tumor lateralization. However, in our study, localization was found to be correct for 6 of 9 patients (66.7%) with negative pituitary MRI results whose lateralization could be determined by IPSS. Thus, these patients were prevented from undergoing total hypophysectomy. In this regard, it is clear that IPSS does have a considerable clinical significance regarding lateralization, especially when pituitary MRI results are negative.

IPSS is generally a safe procedure; most complications reported in the literature have involved inguinal hematomas and thromboembolic complications are rare [17]. No complications developed during or after the sampling procedure in our tertiary center. In particular, probably thanks to a protocol entailing routine administration of heparin, the most feared complications for this procedure, namely thromboembolic complications, were not observed in any cases in our center.

The limitations of our study are due to the low number of patients that could be evaluated as a result of the retrospective study design, as well as the lack of data for postoperative follow-up. The application of IPSS only for patients with insufficient and suspicious imaging results causes selection bias arising from limitations in patient selection and the inability to select a random sample. In addition, since CRH stimulation is applied to all of our patients in the procedure for IPSS, it must be noted that our results may only be applicable to patients for whom CRH stimulation is applied in the course of IPSS. On the other hand, in addition to determining the reliability of IPSS in lateralization in this study, we have evaluated possible factors that may affect the reliability of lateralization, a topic for which limited data are available in the literature, and we have also confirmed the existence of higher rates of accuracy for cases of right-sided lateralization. In addition, we have ascertained that pituitary MRI (when positive) is more reliable than IPSS in the accurate prediction of adenoma lateralization. We are of the opinion that our results represent an important contribution to the literature, to daily practice, and to ensuring correct preoperative determination of tumor localization in patients with CD.

Our study shows that IPSS is a useful, safe application that can guide surgical exploration, especially in cases where imaging possibilities are inadequate. However, if the results are positive, it should be considered that the reliability of pituitary MRI for lateralization may be higher than that of IPSS. Because of the limited accuracy of IPSS in lateralization, the possibility of results of right-sided lateralization predicting the accuracy of IPSS in lateralization should be evaluated further.

## Figures and Tables

**Figure:**
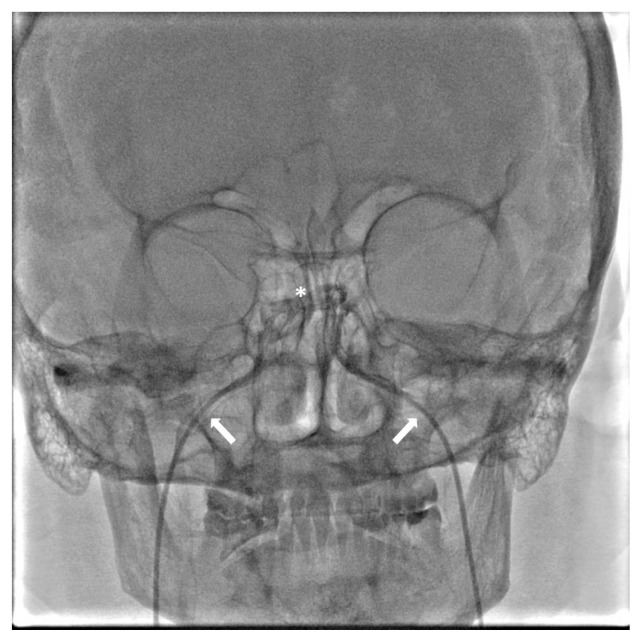
Fluoroscopic image showing the contrast filling both cavernous sinuses, as well as the intercavernous sinus (*). Note that the tips of the diagnostic catheters (arrows) are located in the orifices of the bilateral inferior petrosal sinuses.

**Table 1:** Preoperative endocrinological test results of the patients prior to bilateral inferior petrosal sinus sampling.

Hormonal values	Median (IQR)
Cortizol (6.2–19.4 μg/dL)	21.9 (13.0–25.8)
ACTH (0–46 pg/mL)	65.8 (45.4–96.0)
UFC (10–100 μg/dL)	476 (135.4–795.0)
Midnight serum cortizol (2.3–11.9 μg/dL)	16.7 (12.4–21.1)
DHEAS (35–430 μg/dL)	294 (160.0–392.0)
1 mg DST (μg/dL)	11.7 (8.2–21.3)
Low-dose 2-day DST (μg/dL)	6.7 (4.2–15.7)
High-dose DST (μg/dL)	3.4 (1.7–5.9)

ACTH: adrenocorticotropic hormone, DHEAS: dehydroepiandrosterone sulfate, DST: dexamethasone suppression test, UFC: 24-h urinary free cortisol.

**Table 2:** Lateralization of the adenoma according to bilateral inferior petrosal sinus sampling and ratios.

Case	C/P ratio 0’	C/P ratio max.	Lateralization	Ratio 0’	Ratio max.
1	12.3	9.2	L	2.1	1
2	11.5	15.4	R	7.4	8.7
3	26.6	16	R	5.2	5
4	5	13.2	R	1.3	8.6
5	5.6	20.1	R	11.6	3.4
6	9.4	9.3	R	8.4	7.9
7	6.5	23.1	R	5.8	19.7
8	18.3	13.2	R	8.1	1
9	22.5	23.3	R	18.5	1.4
10	19.3	20.2	L	18.3	14
11	2.3	7.2	L	3	3.3
12	18.9	12.8	R	14.7	6.4
13	14.5	14.7	R	9	12
14	9.7	8.4	R	7.1	3.3
15	2.8	7.9	L	5.1	1.6
16	21.4	11.5	R	2	1
17	30.4	16.4	L	25	13
18	26	9.7	L	10.2	4.4
19	22	17	L	19	12
20	8.2	10	L	6.6	5.7
21	15.2	22.3	R	11.4	18.3
22	10.8	28.7	R	12	22.8
23	6.5	5.8	L	2,3	3
24	4.9	6.5	L	5	4.9
25	21.6	21.5	R	2.4	14.1
26	1.8	8.4	R	1.5	6.4
27	10.1	5.8	R+L	1	1

C/P: central/peripheral, C/P ratio 0: initial C/P ratios for ACTH levels, C/P ratio max: C/P ACTH levels after CRH injection, Ratio 0’: right/left ratios for ACTH levels prior to CRH administration, Ratio max: right/left ratios of ACTH levels following CRH administration.

**Table 3:** Demographic characteristics, preoperative magnetic resonance imaging findings, bilateral inferior petrosal sinus sampling lateralization results and accuracy, surgical approach, histopathological findings, and clinical follow-up results of patients.

		Tumor localization							
Case	S/A	MRI	IPSS (lat)	Actual	Surgery	Pathology	Accuracy of IPSS	Accuracy of MRI	Clinical follow-up results
1	F/28	R, 2.5 mm ma	L	R	RA	CA	False	True	Remission
2	F/51	L, 3 mm ma	R	L	LA	CA	False	True	Remission
3	F/64	L, 6 × 4 mm ma	R	L	TH	G+CA	False	True	Remission
4	F/44	R, 5 mm ma	R	R	RA	CA	True	True	Remission
5	F/38	R, 6 × 3 mm ma	R	R	RA	CA	True	True	Remission
6	F/46	L, 6 × 4 mm+R, HCE	R	R	RHH+LA	NTA	True	False	Remission
7	F/21	HCE	R	R	RHH	CA	True	-	Remission
8	F/53	Empty sella	R	R	RHH	CA	True	-	Remission
9	F/31	R, 5 mm ma	R	R	RA	CA	True	True	Remission, Rec-Reop
10	F/43	L, 6 × 5 mm ma	L	L	LA	CA	True	True	Remission, Rec-GK
11	F/32	L, 5 × 3 mm ma	L	R	LA	NTA	False	False	No Remission, Reop
12	F/65	R,5 × 4+L,3 × 4 mm ma	R	R+L	RA+LA	Bilateral CA	True	True	Remission
13	M/43	R, 6 × 4 mm ma	R	R	RA	CA	True	True	Remission
14	F/32	HCE	R	R+L	RHH	CA	True	-	No Remission, Reop
15	M/36	L, 4 mm ma	L	R+L	LA	CA	True	True	No Remission, Reop
16	F/31	R, 3 mm ma	R	R	RA	CA	True	True	Remission, Rec-Reop
17	F/25	HCE	L	L	LHH	CA	True	-	Remission
18	F/36	HCE	L	R	LHH	NTA	False	-	No Remission, Reop-GK-med
19	F/18	Normal	L	R	LHH	NTA	False	-	No Remission, Reop-med
20	M/41	HCE	L	R	LHH	NTA	False	-	No Remission, Reop-med
21	F/25	L, HCE	R	R	LHH	NTA	True	-	No Remission, Reop-med
22	F/18	R, 6 × 5 mm ma	R	R	RA	CA	True	True	Remission
23	F/46	L,3 mm ma	L	L	LA	CA	True	True	Remission
24	M/26	R, 6 × 3.5 mm ma	L	R	RA	CA	False	True	Remission
25	F/60	L, 6 × 5 mm ma	R	R	LA	GA	True	False	No Remission
26	F/41	HCE	R	R	RHH	CA	True	-	Remission
27	F/48	HCE	-	R+L	TH	Bilateral CA	-	-	Remission

Accuracy: lateralization accuracy of inferior petrosal sinus sampling, CA: corticotroph adenoma, F: female, S/A: sex/age, G: gangliocytoma, GA: gonadotroph adenoma, GK: gamma knife therapy, HCE: heterogeneous contrast enhancement, IPSS: inferior petrosal sinus sampling, L: left, Lat: lateralization, LA: left adenomectomy, LHH: left hemihypophysectomy, M: male, ma: microadenoma, med: medical therapy, MRI: magnetic resonance imaging, N/A: not available, NTA: nontumoral adenohypophyses, R: right, RA: right adenomectomy, Rec: recurrens, Reop: reoperation, RHH: right hemihypophysectomy, TH: total hypophysectomy.

**Table 4:** Patient variables associated with accurate IPSS lateralization prediction.

Variable	PPV present (%)	PPV absent (%)	p value
Response pattern to CRH			
Consistent lateralization	15 (83.3)	7 (87.5)	1.00
Others*	3 (16.7)	1 (12.5)	
Side of lateralization			
Right	14 (77.8)	2 (25.0)	**0.026**
Left	4 (22.2)	6 (75.0)	
Peak interpetrosal gradient ratio	11.8 (7.1–18.3)	5.9 (4.1–9.4)	0.107
Peak interpetrosal gradient ratio time			
Before CRH administration	10 (55.6)	6 (75.0)	0.420
After CRH administration	8 (44.4)	2 (25.0)	
Age	40 (31–46)	34 (27–46)	0.656
Sex, female	16 (88.9)	6 (75.0)	0.563
MRI positivity	10 (83.3)	4 (80.0)	1.00
Ratio 0’	8.2 (2.4–12.08)	5.9 (4.0–8.8)	0.579
Ratio max.	7.1 (3.0–14.0)	4.9 (3.8–7.2)	0.404

CRH: corticotropin-releasing hormone, IPSS: inferior petrosal sinus sampling, MRI: magnetic resonance imaging, Ratio 0’: right/left ratios for ACTH levels prior to CRH administration, Ratio max: right/left ratios of ACTH levels following CRH administration,

* Others: lateralization only before CRH administration and Lateralization only after CRH administration.

## References

[1] Fleseriu M Auchus R Bancos I Ben-Shlomo A Bertherat J Consensus on diagnosis and management of Cushing’s disease: a guideline update The Lancet Diabetes & Endocrinology 2021 9 12 847 875 10.1016/S2213-8587(21)00235-7 34687601 PMC8743006

[2] Melmed S Pituitary-tumor endocrinopathies New England Journal of Medicine 2020 382 10 937 950 10.1056/NEJMra1810772 32130815

[3] Wind JJ Lonser RR Nieman LK DeVroom HL Chang R The lateralization accuracy of inferior petrosal sinus sampling in 501 patients with Cushing’s disease The Journal of Clinical Endocrinology & Metabolism 2013 98 6 2285 2293 10.1210/jc.2012-3943 23553862 PMC3667263

[4] Oldfield EH Doppman JL Nieman LK Chrousos GP Miller DL Petrosal sinus sampling with and without corticotropin-releasing hormone for the differential diagnosis of Cushing’s syndrome New England Journal of Medicine 1991 325 13 897 905 10.1056/NEJM199109263251301 1652686

[5] Lefournier V Martinie M Vasdev A Bessou P Passagia J-G Accuracy of bilateral inferior petrosal or cavernous sinuses sampling in predicting the lateralization of Cushing’s disease pituitary microadenoma: influence of catheter position and anatomy of venous drainage The Journal of Clinical Endocrinology & Metabolism 2003 88 1 196 203 10.1210/jc.2002-020374 12519852

[6] Nieman LK Biller BM Findling JW Newell-Price J Savage MO The diagnosis of Cushing’s syndrome: an endocrine society clinical practice guideline The Journal of Clinical Endocrinology & Metabolism 2008 93 5 1526 1540 10.1210/jc.2008-0125 18334580 PMC2386281

[7] Nieman LK Biller BM Findling JW Murad MH Newell-Price J Treatment of Cushing’s syndrome: an endocrine society clinical practice guideline The Journal of Clinical Endocrinology & Metabolism 2015 100 8 2807 2831 10.1210/jc.2015-1818 26222757 PMC4525003

[8] Doppman JL Chang R Oldfield EH Chrousos G Stratakis CA The hypoplastic inferior petrosal sinus: a potential source of false-negative results in petrosal sampling for Cushing’s disease The Journal of Clinical Endocrinology & Metabolism 1999 84 2 533 540 10.1210/jcem.84.2.5475 10022412

[9] Mamelak AN Dowd CF Tyrrell JB McDonald JF Wilson CB Venous angiography is needed to interpret inferior petrosal sinus and cavernous sinus sampling data for lateralizing adrenocorticotropin-secreting adenomas The Journal of Clinical Endocrinology & Metabolism 1996 81 2 475 481 10.1210/jcem.81.2.8636253 8636253

[10] Colao A Faggiano A Pivonello R Giraldi FP Cavagnini F Inferior petrosal sinus sampling in the differential diagnosis of Cushing’s syndrome: results of an Italian multicenter study European Journal of Endocrinology 2001 144 5 499 507 10.1530/eje.0.1440499 11331216

[11] Deipolyi A Bailin A Hirsch JA Walker TG Oklu R Bilateral inferior petrosal sinus sampling: experience in 327 patients Journal of Neurointerventional Surgery 2017 9 2 196 199 10.1136/neurintsurg-2015-012164 26880723

[12] Castinetti F Morange I Dufour H Jaquet P Conte-Devolx B Desmopressin test during petrosal sinus sampling: a valuable tool to discriminate pituitary or ectopic ACTH-dependent Cushing’s syndrome European Journal of Endocrinology 2007 157 3 271 277 10.1530/EJE-07-0215 17766708

[13] Feng M Liu Z Liu X Zhang X Bao X Tumour lateralization in Cushing’s disease by inferior petrosal sinus sampling with desmopressin Clinical Endocrinology 2018 88 2 251 257 10.1111/cen.13505 29080355

[14] Yanovski JA Nieman LK Doppman JL Chrousos GP Wilder RL Plasma levels of corticotropin-releasing hormone in the inferior petrosal sinuses of healthy volunteers, patients with Cushing’s syndrome, and patients with pseudo-Cushing states The Journal of Clinical Endocrinology & Metabolism 1998 83 5 1485 1488 10.1210/jcem.83.5.4766 9589643

[15] Buchfelder M Nistor R Fahlbusch R Huk WJ The accuracy of CT and MR evaluation of the sella turcica for detection of adrenocorticotropic hormone-secreting adenomas in Cushing disease American Journal of Neuroradiology 1993 14 5 1183 1190 8237701 PMC8332751

[16] Pereira CA Ferreira L Amaral C Alves V Xavier J Diagnostic accuracy of bilateral inferior Petrosal sinus sampling: the experience of a tertiary Centre Experimental and Clinical Endocrinology & Diabetes 2021 129 02 126 130 10.1055/a-0981-5973 31426111

[17] Deipolyi A Karaosmanoğlu A Habito C Brannan S Wicky S The role of bilateral inferior petrosal sinus sampling in the diagnostic evaluation of Cushing disease Diagnostic and Interventional Radiology 2011 18 1 132 138 10.4261/1305-3825.DIR.4279-11.0 21348009

